# Brief Report: Mindfulness Training for Chinese Adolescents with Autism Spectrum Disorder and Their Parents in Hong Kong

**DOI:** 10.1007/s10803-020-04729-4

**Published:** 2021-01-23

**Authors:** Ryan Yuk Fai Ho, Dexing Zhang, Stanley Kam Chung Chan, Tiffany Ting Gao, Eric Kam Pui Lee, Herman Hay Ming Lo, Peter Au Yeung, Kelly Yee Ching Lai, Susan M. Bögels, Esther I. de Bruin, Samuel Yeung Shan Wong

**Affiliations:** 1 JC School of Public Health and Primary Care, The Chinese University of Hong Kong, Hong Kong, China; 2 Thomas Jing Centre for Mindfulness Research and Training, The Chinese University of Hong Kong, Hong Kong, China; 3 Present Address: New Life Psychiatric Rehabilitation Association, Hong Kong, China; 4 Department of Applied Social Sciences, The Hong Kong Polytechnic University, Kowloon, Hong Kong, China; 5 Heep Hong Society, Hong Kong, China; 6 Research Institute of Child Development and Education, The University of Amsterdam, Amsterdam, Netherlands; 7 Department of Psychiatry, The Chinese University of Hong Kong, Hong Kong, China

**Keywords:** Autism spectrum disorder, MYmind, Feasibility, Effectiveness

## Abstract

This study investigated the feasibility and preliminary effectiveness of a concurrent mindfulness program (MYmind) on Chinese adolescents with autism spectrum disorder and their parents in Hong Kong, China using a randomized controlled trial with a waitlist control group. Results showed the study had 80% compliance rate, 0% dropout rate, and 89% response rate. Between-group comparisons showed mindfulness had trend effects on parent’s rumination (*g* = 1.16), mindful parenting (*d* = 0.6), parenting style (*d* = 0.59), and parenting stress (*d* = 0.5). The study demonstrated the feasibility of the MYmind program in the Chinese context. A larger trial with longer follow-up period is suggested to better examine the effect of mindfulness on adolescents with ASD and their parents.

## Introduction

Studies have shown autism spectrum disorder (ASD) has an increasing prevalence in the last decade (Matson and Kozlowski 2011; Polanczyk et al. 2015). In the Chinese population, the pooled prevalence of ASD is 26.6 per 10,000 (95% Confidence Interval: 18.5, 34.6; Sun et al. 2013). ASD is one of the common neurodevelopmental disorders observed among adolescents. Besides impaired social communication and restricted, repetitive patterns of behaviour (American Psychiatric Association 2013), adolescents with ASD exhibit externalizing and internalizing problems (Bauminger et al. 2010), attention problems (Bauminger et al. 2010), as well as deficits in executive function (Margari et al. 2016). They experience difficulties in behavioural regulation (e.g. controlling impulses, shifting attention, and regulating emotion) and metacognition (e.g. implementing problem-solving strategies, working memory, planning events, organizing materials, and self-monitoring). As it concerns the many difficulties that adolescents with ASD have, treatments are needed to improve their competence and functions in daily life, as well as comorbid emotional problems.

Currently, applied behavioural analysis (ABA), social intervention, CBT, and medication are the most common treatments for adolescents with ASD. They are shown to have some positive impacts on social functioning, anxiety, and behavioural problems. However, there are certain limitations to each treatment. For example, ABA is intensive and requires long-term participation to optimize the outcomes. Also, most of the ABA effectiveness studies are conducted on young children with ASD, instead of adolescents with ASD (Reichow and Volkmar 2010). Social skill interventions are shown to have a small treatment effect (Gates et al. 2017) and might lead to increased anxiety after training (Swain et al. 2015). Cognitive behavioural therapy (CBT) is effective in treating comorbid anxiety of individuals with ASD (Sukhodolsky et al. 2013), but not ASD itself. Medications have adverse side-effects and similar effectiveness compared to placebo (Reddihough et al. 2019; Yatawara et al. 2015). As it concerns the shortcomings in current treatments, further research is required to explore new treatments.

Mindfulness training could be a potential treatment for adolescents with ASD. Recently, a mindfulness-based intervention (MBI), called the MYmind program, was developed for adolescents, who have ASD without having intellectual disability or borderline intelligence, and their parents. It consists of 9 weekly 1.5-h mindfulness-training sessions for adolescents and parents separately. There are several reasons to suggest the MYmind program has beneficial effects on adolescents with ASD. Firstly, in consideration of ASD symptoms, mindfulness involves focusing on the present moment, which includes moments of having interaction with others. It helps adolescents with ASD pay attention to social cues during interaction with others and give appropriate responses. Secondly, in consideration of executive function, mindfulness involves mind–body exercise, which allows adolescents with ASD to shift attention and monitor the interaction among mind, body, and behaviours, hence foster self-control of emotion and behaviour (Chan et al. 2013). Also, studies have shown practising mindfulness could protect against proactive interference and promote insightful thinking (Greenberg et al. 2019; Ren et al. 2011), which could in turn improve metacognition (e.g. working memory and problem-solving) of adolescents with ASD. Thirdly, in consideration of attention problems, mindfulness meditation helps adolescents with ASD stay present by bringing attention back to breath whenever get distracted. Fourthly, in consideration of externalizing and internalizing problems, mindfulness helps adolescents with ASD promote self-reflection (Marcovitch et al. 2008), which could facilitate positive behavioural changes. Moreover, long-term meditation training could reduce amygdala reactivity (Kral et al. 2018), which suggests improved emotional responses of adolescents with ASD toward stress. Fifthly, the MYmind program involves mindfulness training for parents. It allows parents to establish a positive parenting style by being thoughtful and empathic, which in turn decreases their children's problem behaviours (Singh et al. 2006, 2014). Also, previous studies have shown parents of children with ASD experience higher levels of stress, rumination, and poorer well-being than parents of children with typical development (Bonis 2016; Carpita et al. 2019; Giallo et al. 2013; Lai et al. 2015; Wang et al. 2013). Providing mindfulness training could help them alleviate these problems and reduce their burdens.

In regard to previous MBI studies to adolescents with ASD and their parents, many of them focused on providing mindfulness training to parents, and a few of them focused on providing mindfulness training to adolescents with ASD. To our knowledge, only a limited number of studies had examined the use of MBI on both adolescents with ASD and parents at the same time. For the MYmind program, three prior studies explored its effectiveness on adolescents with ASD and parents with pre-post-follow up design (de Bruin et al. 2015; Ridderinkhof et al. 2017; Salem-Guirgis et al. 2019). Various measurements were used among the studies and consistent findings were found that adolescents with ASD had improved social responsiveness, while parents had improved mindful parenting skills after the program. Other positive findings among the studies showed that mindfulness training had beneficial effects on adolescents with ASD (in terms of attention problems, externalizing and internalizing problems) and parents (in terms of parenting style, well-being, and parenting stress) (de Bruin et al. 2015; Ridderinkhof et al. 2017; Salem-Guirgis et al. 2019). Apart from quantitative studies, a qualitative study was conducted to interview adolescents with ASD and their parents about the experience of participating in the MYmind program (Ridderinkhof et al. 2019). Results showed the program was positively rated and both adolescents with ASD and parents reported positive self-changes in social and emotional aspects, such as attuning to others and being calm.

Despite the positive results found in the previous MYmind studies, the effects of mindfulness on adolescents with ASD and their parents need to be further investigated because none of the previous studies has a control group to compare with the intervention group (de Bruin et al. 2015; Ridderinkhof et al. 2017; Salem-Guirgis et al. 2019). Since there is a lack of studies investigating the effectiveness of MBI on families of adolescents with ASD in the Chinese context, the current study represents the first pilot randomized controlled trial to evaluate the feasibility and preliminary effectiveness of the MBI (MYmind program) on Chinese adolescents with ASD and their parents in Hong Kong, China. In light of the prior findings (de Bruin et al. 2015; Ridderinkhof et al. 2017; Salem-Guirgis et al. 2019), we not only aim to explore the effectiveness of mindfulness training on Chinese adolescents with ASD (in terms of social responsiveness, attention problems, externalizing and internalizing problems) and their parents (in terms of parenting stress, parenting style, mindful parenting, and mental well-being), but also to examine whether mindfulness training could improve executive function of adolescents with ASD and reduce rumination of parents. We hypothesized that the MYmind program was feasible and effective to adolescents with ASD (reduced attention problems, externalizing and internalizing problems, and improved social responsiveness and executive function) and parents (reduced rumination and parenting stress, and improved mindful parenting skills, mental well-being, and parenting style) when compared to a waitlist control group.

## Methods

### Participants

Adolescents with ASD and their parents were recruited through community-based seminars and social media. The recruitment period was from April 2017 to June 2018. The inclusion criteria were: (1) 10 to 18-year-old adolescent who was diagnosed with ASD by a psychologist or psychiatrist according to the Diagnostic and Statistical Manual of Mental Disorders DSM-V (American Psychiatric Association 2013), (2) at least one of the parents agreed to participate in the MYmind program. The exclusion criteria were: (1) either adolescent or parent was unable to understand or speak in Chinese as the MYmind program was conducted in Chinese, (2) adolescent had borderline intelligence or intellectual disability, (3) adolescent had a medical condition rendering himself/herself to be incapable to participate in the study, (4) either adolescent or parent was unwilling or unable to give consent.

Parents were informed of the study’s purpose and procedure in detail by a research assistant. A consent form was signed before the study participation. The study was approved by the Joint Chinese University of Hong Kong—New Territories East Cluster Clinical Research Ethics Committee (The Joint CUHK-NTEC CREC) and was registered in Chinese Clinical Trial Registry with a reference number of ChiCTR-IPR-17011233**.**

### Procedure

**Fig. 1:**
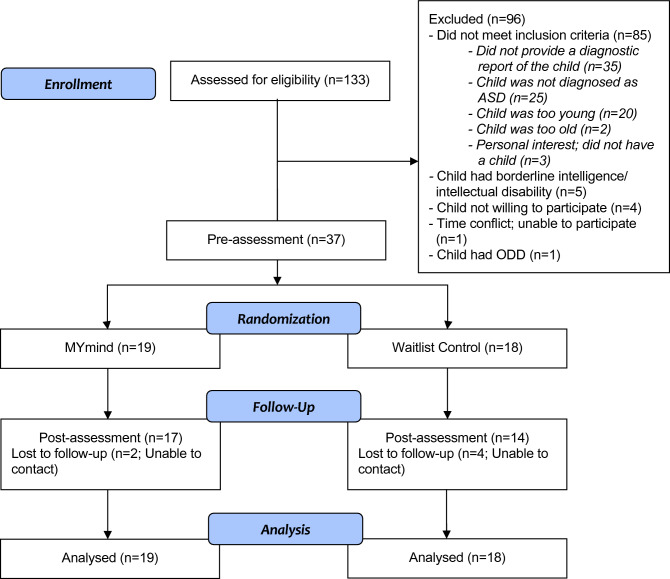
CONSORT diagram

This study was a randomized controlled trial. Participating families were randomized into either (i) immediate MYmind program (6 to 12 children per group) or (ii) waitlist-control group, which they received the MYmind program 9 weeks later. The MYmind program was arranged in summer vacation. Pre-specified questionnaires were self-administrated by parents in both groups before and after the 9-week MYmind program (See Fig. 1). In addition, parents in the MYmind group filled in the evaluation survey of the program.

The MYmind program followed the protocol which had been used in previous studies (de Bruin et al. 2015; Ridderinkhof et al. 2017; Salem-Guirgis et al. 2019). The concurrent intervention included 9 weekly 90-min mindfulness training sessions for adolescents with ASD and their respective parents. In both of the adolescent’s and the parent’s training, each session consisted of theoretical knowledge, mindfulness exercises, experience sharing, and homework assignments. Adolescents learned to improve concentration, enhance mental emotional capacity and the ability to relate to others; parents learned to reduce stress and apply mindfulness in parenting to establish positive parent–child interaction. Mindfulness exercises included various types of meditation (breathing, body, sound, thought, walking) and yoga practice; these exercises were based on the mindfulness-based cognitive therapy (MBCT) and the mindfulness-based stress reduction program (MBSR) (Segal et al. 2012; Kabat-Zinn 1982). Homework assignments consisted of reading handouts, listening to instruction audios to practice mindfulness exercises, and diary registrations. The intervention was offered by trained mindfulness teachers who were either educational/clinical psychologists or social workers and had (1) experience in treating adolescents with special needs and their families; (2) undergone an 8-week mindfulness-based stress reduction (MBSR) or mindfulness-based cognitive therapy (MBCT) program; (3) completed a 4-day MYmind advanced teacher training by one of the developers of the MYmind program, Professor Bögels from the University of Amsterdam; (4) completed a 6 or 7-day MBCT/MBSR teacher training; (5) at least 4-year experience of own mindfulness practice.

### Randomization

*Micros*oft Excel was used to perform simple randomization by an independent research assistant. The first step of the approach was to create a column and arrange the treatment (MYmind group or waitlist control group) in a systematic order. And then, created a second column and generated random numbers using the RAND() function. At last, sorted the second column filled with random numbers in ascending order, which produced a list of treatments in random order.

### Study Blinding

Due to the study design, it was not possible to blind the participants and mindfulness instructors. However, the research assistants who collected and analysed the data were blinded from the treatment allocation.

### Sample Size

In Browne’s research (1995), the recommended pilot study sample size was 30. To estimate the sample size of a parallel-group trial, Julious’s study (2005) suggested a sample size of 12 per group. In our study, we aimed to recruit 30–40 families as a rule of thumb.

### Measurements

#### Primary Outcomes

The primary outcomes were feasibility and acceptability of the MBI (MYmind program) as measured by the following: (1) Recruitment rate; (2) Compliance rate reflected by the attendance rate of the nine mindfulness training sessions; (3) Retention rate reflected by the dropout rate and the response rate (number of parents who completed the post-assessment divided by the total number of parents) of the MYmind group; (4) Program evaluation consisting of 4 questions reported on a 10-point scale: (a) easiness of the program contents (1 = very difficult; 10 = very easy), (b) helpfulness to parents (1 = totally not helpful; 10 = very helpful), (c) helpfulness to adolescents (1 = totally not helpful; 10 = very helpful), and (d) satisfaction of the program (1 = totally unsatisfied; 10 = very satisfied). To indicate the program was acceptable, all the questions had to achieve an average score of 5 or above.

### Secondary Outcomes for Adolescent

#### Social Responsiveness Scale (SRS)

The Social Responsiveness Scale (SRS) is developed to identify the presence of autistic behaviours and the extent of social impairment of 4–18 years old children (Constantino and Gruber 2005). It has 65 items rated from 1 to 4 for “not true” to “true”. Parents are instructed to rate the items based on the child’s behaviours in the past six months. The SRS has been used in the previous MYmind studies (de Bruin et al. 2015; Ridderinkhof et al. 2017; Salem-Guirgis et al. 2019) and has been validated in Chinese version with an internal consistency of 0.87–0.92 and test–retest reliability of 0.81–0.94 (Cen et al. 2017). The internal reliability of SRS total score in the current sample was 0.94.

#### The Child Behaviour Checklist (CBCL)

The Child Behaviour Checklist (CBCL) is widely used to identify problematic behaviours of children aged 6–18 years (CBCL/6-18) (Achenbach 1999). The checklist is reported by parents who see the children in home-like settings. Parents are instructed to rate the items based on the child’s behaviours in the past six months. The responses are recorded on a Likert scale from 0 (not true) to 2 (very true or often true). Higher scores indicate greater problems. The CBCL has shown high cross-cultural consistency and the Chinese version has been validated (Crijnen et al. 1999; Dedrick et al. 2008). Also, the CBCL has been used in the previous MYmind trial (Ridderinkhof et al. 2017). In this study, it was reported by two broadband syndrome scales (externalizing problems and internalizing problems) and the attention problems subscale. The internal reliability of internalizing problems score, externalizing problems score, and attention problems score were 0.86–0.90, 0.89–0.91, and 0.78 respectively.

#### The Behaviour Rating Inventory of Executive Function (BRIEF)

The BRIEF measures children’s executive function and is reported by parents, who rate the 86 items based on their children’s behaviours in the past six months (Gioia et al. 2000, 2002). Higher scores indicate poorer outcomes. The BRIEF has been used in ASD population (Chan et al. 2009; Gilotty et al. 2002; Kenworthy et al. 2009). It has had good test–retest reliability ranging from 0.68 to 0.89 and internal consistency ranging from 0.74 to 0.96 in the Chinese version (Qian and Wang 2007). In the study, the BRIEF was reported by two index scores (behavioural regulation index and metacognition index) and a total score (global executive composite).

### Secondary Outcomes for Parent

#### Parenting Stress Index (PSI)

Parenting Stress Index (PSI) is a 36-item questionnaire measuring parenting stress (Abidin 1995). Three subscale scores are computed in addition to a total score: Parental Distress (PD), Parent–Child Dysfunctional Interaction (PCDI), and Difficult Child (DC). Higher scores indicate higher parenting stress. The PSI has been used in the previous MYmind studies (de Bruin et al. 2015; Ridderinkhof et al. 2017), and the Chinese version has been validated and used in Hong Kong (Lam 1999; Tam et al. 1994). The PSI has had a reliability coefficient of 0.93 and it could discriminate “high-stress” respondents from “low-stress” respondents with 93% overall accuracy (Tam et al. 1994). The internal reliability of PSI total score in the current sample was 0.91.

#### Parenting Scale (PS)

The PS is a measure of dysfunctional parenting in discipline situations (Arnold et al. 1993). It has been used in the previous MYmind studies (de Bruin et al. 2015; Ridderinkhof et al. 2017). It has 3 domain scales: laxness, over-reactivity, and hostility (Rhoades et al. 2007). The items are scored on a 7-point scale with higher scores indicating more dysfunctional parenting. Parents are instructed to rate the items based on the parenting behaviours in the past two months. The PS has had adequate internal reliability in the Chinese version (Leung et al. 2003). The internal reliability of PS total score in the current sample was 0.38; laxness scale was 0.49; over-reactivity scale was 0.76; hostility scale was 0.83.

#### The Interpersonal Mindfulness in Parenting (IM-P)

The IM-P assesses parents’ quality of mindfulness in parenting interaction (Duncan 2007). It has been used in the previous MYmind studies (de Bruin et al. 2015; Ridderinkhof et al. 2017; Salem-Guirgis et al. 2019). The Chinese validated version of the IM-P includes 4 subscales: (1) compassion for child; (2) non-judgmental acceptance in parenting; (3) emotional awareness in parenting; (4) listening with full attention (Lo et al. 2018). The Chinese version of the IM-P has had an overall internal consistency of 0.85, and the four subscales have had internal consistencies in a range of 0.70–0.84 (Lo et al. 2018). In the current sample, the overall internal consistency was 0.89 and those of four subscales were ranged from 0.66 to 0.82.

#### WHO-5 Well-being Index (WHO-5)

The WHO-5 consists of five positively worded items that reflect the presence or absence of well-being rather than depressive symptomatology: (1) I have felt cheerful and in good spirits, (2) I have felt calm and relaxed, (3) I have felt active and vigorous, (4) I woke up feeling fresh and rested, and (5) My daily life has been filled with things that interest me (Bech et al. 2003). The responses are recorded on a 6-point scale ranging from all of the time (5 points) to at no time (0 points). Parents are asked to report the items based on the experience in the last 2 weeks. The WHO-5 has had a good construct validity (Topp et al. 2015) and has been used in the previous MYmind studies (de Bruin et al. 2015; Ridderinkhof et al. 2017). The WHO-5 in the Chinese version has had an internal consistency of 0.84 (Volinn et al. 2010). The internal reliability of WHO-5 total score in the current sample was 0.94.

#### The Rumination Response Scale (RRS)

The RRS is a self-report measure of one’s responses to depressed mood (Treynor et al. 2003). Items are scored on 4-point Likert scales, resulting in a possible range of scores from 22 to 88. The RRS has possessed a good internal consistency of 0.85 in the validated Chinese version (Yang et al. 2009). The internal reliability of RRS total score in the current sample was 0.93.

### Data Analyses

#### Data Collection and Management

All the data were collected by trained research assistants. The questionnaires were sent through mails, completed by parents, and returned with an envelope provided. Demographical data, e.g., age, gender, education, marital status, income, and employment status were collected during recruitment (Table 1). To increase the compliance rate of the study, text reminders of class attendance were sent out to parents’ mobile chat group. Parents were informed of their assessment results after the completion of the whole study.

**Table 1:** Demographic information of the participants

			MYmind (n = 19)	Control (n = 18)	Total (n = 37)
Parent	Age* (Mean, SD)		49.1 (5.4)	44.1 (5.5)	46.5 (6.0)
	Female		68%	83%	76%
	Education*	High school	32%	33%	32%
		Diploma	0%	28%	14%
		College or above	68%	39%	54%
	Marriage	Married	84%	94%	89%
		Re-married	11%	0	6%
		Divorced/separated	5%	6%	5%
	Income	< HK$ 5000	6%	0%	3%
		$5001–10,000	0	6%	3%
		$10,001–20,000	17%	17%	17%
		$20,001–30,000	6%	17%	11%
		$30,001–40,000	18%	28%	23%
		> $40,000	53%	33%	43%
	No. family members (Mean, SD)		3.8 (0.9)	3.7 (0.6)	3.7 (0.7)
		2	6%	6%	6%
		3	31%	18%	24%
		4	44%	76%	61%
		5	19%	0%	9%
Child	Age (Mean, SD)		13.7 (2.3)	12.5 (2.1)	13.0 (2.3)
	Male		68%	83%	76%
	Year of diagnosis		8.2 (3.6)	6.2 (3.6)	7.3 (4.3)

*p−value was significant, p < 0.05

### Statistical Methods

Data were analyzed by using STATA 12 (StataCorp, 2011). Cronbach’s α was used to measure the internal consistency of the study measurements. Mean, standard deviation, frequency, and percentage were used for data description. Independent samples t-tests and chi-squared tests were conducted to compare the demographic data between the two groups (See Table 1). For secondary outcomes (See Tables 2 and 3), Cohen’s *d* was used to measure the effect sizes of within-group comparison and between-group comparison. Levene's test was used to assess the equality of standard deviations. If significant differences were detected, Glass’s delta was used to measure the effect sizes instead of Cohen’s *d*. The cut-off points of 0.2, 0.5, and 0.8 were used to denote small, medium, and large effect sizes. In addition, multilevel mixed-effects regression with time (pre-assessment and post-assessment) as the within-subjects variable and group (MYmind group and waitlist control group) as the between-subjects variable, was used to detect the effects of time, group, and time x group interactions on each of the outcome measures with adjustment of parent’s age and education. The Holm–Bonferroni sequential correction was used to counteract the problem of multiple comparisons (Aickin and Gensler 1996; Holm 1979).

**Table 2:** Adolescent’s within-group and between-group comparison of secondary outcomes in MYmind group and waitlist control group in the pre- and the post-assessment

Measure			MYmind Mean (SD)	Cohen's d	Control Mean (SD)	Cohen's d	Cohen's d (between group)
SRS	Total score	Pre	102.9 (29.1)	0.42	103.2 (23.7)	0.63	0.01
		Post	90.9 (28.4)		88.2 (23.8)		
CBCL	Attention problems	Pre	65.4 (8.9)	0.26	67.1 (10.0)	0.14	0.21
		Post	63.0 (9.6)		65.4 (13.4)		
	Internalizing problems	Pre	61.5 (9.3)	0.27	64.1 (7.1)	0.52^a^	0.14
		Post	58.9 (9.7)		60.4 (11.8)		
	Externalizing problems	Pre	59.6 (8.4)	0.34	60.3 (7.8)	0.26	0.29
		Post	56.9 (7.3)		58.2 (8.5)		
BRIEF	BRI	Pre	70.1 (13.5)	0.03	71.6 (9.9)	0.22	0.12
		Post	70.4 (10.1)		69.2 (11.5)		
	MI	Pre	66.5 (11.5)	0.08	63.3 (7.7)	0.19	0.38
		Post	65.6 (11.0)		61.6 (9.8)		
	GEC	Pre	69.2 (11.7)	0.05	67.6 (7.2)	0.24	0.31
		Post	68.6 (10.3)		65.5 (9.8)		

The mixed-effects regression was used to measure the time × group interaction effect of each outcome measure with adjustment of parent’s age and education

All outcomes’ p values were > 0.05

*SRS* social responsiveness scale, *CBCL* child behaviour checklist, *BRIEF* behaviour rating inventory of executive function, *BRI* behavioural regulation index, *MI* metacognition index, *GEC* global executive composite

^a^Effect sizes were measured by Glass’s delta

**Table 3:** Parent’s within-group and between-group comparison of secondary outcomes in MYmind group and waitlist control group in the pre- and the post-assessment

Measure			MYmind Mean (SD)	Cohen's d	Control Mean (SD)	Cohen's d	Cohen's d (between group)
PSI	Total	Pre	113.7 (18.9)	0.32	110.6 (21.9)	0.34	0.21
		Post	107.6 (19.1)		103.5 (20.2)		
	PD	Pre	34.5 (6.7)	0.22	36.7 (10.7)	0.30	0.09
		Post	33.0 (6.9)		33.7 (9.2)		
	PCDI	Pre	38.8 (7.5)	0.12	34.2 (8.5)	0.03	0.50
		Post	37.9 (8.0)		34.4 (5.8)		
	DC	Pre	41.9 (7.5)	0.50	39.3 (6.8)	0.42	0.22
		Post	38.0 (8.2)		36.3 (7.5)		
PS	Total	Pre	115.0 (10.3)	0.16	114.0 (11.9)	0.01	0.06
		Post	113.3 (10.7)		113.9 (9.7)		
	Laxness	Pre	16.5 (4.9)	0.60	17.4 (3.9)	0.41	0.13
		Post	19.1 (3.7)		18.7 (2.3)		
	Over-reactivity	Pre	20.7 (6.2)	0.56	19.9 (5.5)	0.11	0.59
		Post	17.5 (5.1)		20.5 (5.0)		
	Hostility	Pre	6.9 (4.2)	0.19	6.2 (4.4)	0.18	0.21
		Post	6.2 (3.0)		6.9 (3.5)		
IM-P	Total	Pre	78.4 (11.0)	0.35	77.3 (13.0)	0.07	0.53
		Post	81.9 (9.1)		76.4 (11.5)		
	Listening with full attention	Pre	13.3 (2.1)	0.38	12.9 (3.1)	0.17	0.29
		Post	14.1 (2.1)		13.4 (2.7)		
	Emotional awareness	Pre	20.5 (3.2)	0.16	19.8 (3.5)	0.32	0.60
		Post	21.1 (4.2)		18.5 (4.5)		
	Non-judgmental acceptance	Pre	17.8 (4.4)	0.46	19.1 (5.1)	0.28	0.44
		Post	19.4 (2.3)		17.7 (4.9)		
	Compassion	Pre	26.8 (3.9)	0.15	26.2 (4.7)	0.14	0.14
		Post	27.4 (4.2)		26.8 (4.1)		
WHO-5	Total	Pre	11.4 (5.2)	0.31	10.4 (6.8)	0.34	0.13
		Post	13.1 (5.9)		12.4 (4.7)		
RRS	Total	Pre	40.9 (8.5)	0.61	41.4 (11.1)	0.27	1.16^a^
		Post	36.0 (7.5)		44.7 (13.4)		

The mixed-effects regression was used to measure the time x group interaction effect of each outcome measure with adjustment of parent’s age and education

All outcomes’ p values were > 0.05

*PSI* parenting stress index, *PS* parenting scale, *IM-P* interpersonal mindfulness in parenting, *WHO-5* WHO-5 well-being index, *RRS* rumination response scale, *PD* parental distress, *PCDI* parent–child dysfunctional interaction, *DC* difficult child

^a^Effect sizes were measured by Glass’s delta

## Results

A total of 133 families registered for the study (See Fig. 1). In the screening process, 90 (68%) families were not eligible for the study. The major reasons were parents were unable to provide a diagnostic report of their child, and adolescents either did not have a diagnosis of ASD or were too young to join the program. There was 1 family excluded from the study because the adolescent had both ASD and oppositional defiant disorder (ODD). In the end, 37 out of 42 (88%) eligible families were included in the study; 19 families were randomised to the MYmind group and 18 families were randomised to the waitlist control group. Seventeen (89%) parents in the MYmind group and 14 (78%) parents in the waitlist control group completed the post-assessment.

### Demographic Characteristics

The average age of parents was 46.0 (*SD* = 6.0) years old (See Table 1). There were 76% of the parents who participated the MYmind program were mothers, 54% of the parents had a college degree or above, 89% of the parents were married, 66% of the parents had a monthly family income at HK$30001 (about US$ 3800) or above. The average number of family members was 3.7 (*SD* = 0.7). The average age of the participating adolescents was 13.0 (*SD* = 2.3) years old, 76% of them were boys. The participating adolescents received their diagnoses 7.3 (*SD* = 4.3) years ago. The demographic characteristics of parents in both the MYmind group and the waitlist control group were balanced at baseline except MYmind group had an older age (49.1 (*SD* = 5.4) vs. 44.1 (5.5), *p* = 0.017) and higher educational level (college or above: 68% vs. 39%, diploma: 0% vs. 28%, and high school: 32% vs. 33%, *p* = 0.034).

### Attendance and Course Evaluation (Primary Outcomes)

There were 15 (78.9%) parents and 15 (78.9%) adolescents who attended at least 6 out of 9 sessions respectively. On average, parents attended 7.16 (*SD* = 2.03) sessions, and adolescents attended 7.21 (*SD* = 1.87) sessions. The overall attendance rate was high, which was 80%. The reasons for session absence included adolescent or parent sickness and time clashes with personal activities. Adolescent’s attendance rate was higher than that of parent’s because sometimes other family members brought the adolescents to class instead of parents.

The program evaluation results (N = 11) showed that the average score of the perceived degree of easiness of the program contents was 5.91 (*SD* = 1.64) out of 10 (1 = very difficult; 10 = very easy); there were 9 (81.8%) parents who scored 5 or above. The average score of the perceived helpfulness to parents was 5.91 (*SD* = 2.21) out of 10 (1 = totally not helpful; 10 = very helpful); there were 9 (81.8%) parents who scored 5 or above. The average score of the perceived helpfulness to adolescents was 4.73 (*SD* = 2.24) out of 10 (1 = totally not helpful; 10 = very helpful); there were 6 (54.5%) parents who scored 5 or above. The average satisfaction score of the program was 6.55 (*SD* = 2.25) out of 10 (1 = totally unsatisfied; 10 = very satisfied); there were 8 (72.7%) parents who scored 5 or above.

### Quantitative Results (Secondary Outcomes)

In the within-group comparison of adolescent’s secondary outcomes (See Table 2), both the MYmind and the waitlist control groups showed improving trends in SRS with effect sizes of 0.42 and 0.63 respectively, and in CBCL subscales with effect sizes ranging from 0.26 to 0.34 and from 0.14 to 0.52 respectively. For BRIEF subscales, the changes were trivial in the MYmind group with effect sizes ranged from 0.03 to 0.08, while the waitlist control group had effect sizes ranged from 0.19 to 0.24.

In the within-group comparison of parent’s secondary outcomes (See Table 3), both the MYmind and the waitlist control groups showed improving trends in PSI subscales with effect sizes ranged from 0.12 to 0.50 and from 0.03 to 0.42 respectively, as well as in WHO-5 with effect sizes of 0.31 and 0.34 respectively. For PS subscales, an increase in laxness subscale was found in both the MYmind group and the waitlist control group with effect sizes of 0.6 and 0.41 respectively. For IM-P subscales, the MYmind group showed improvements with effect sizes ranged from 0.15 to 0.46, while the waitlist control group showed improvements to a small extent in the two subscales (listening with full attention, compassion) with effect sizes of 0.17 and 0.14 respectively. For RRS, the MYmind group showed improvement with an effect size of 0.61, while the waitlist control group showed a decline with an effect size of 0.27. Between-group effect sizes of 0.5, 0.59, 0.6, and 1.16 were found in parent–child dysfunctional interaction subscale of PSI, over-reactivity subscale of PS, emotional awareness subscale of IM-P, and RRS respectively.

## Discussion

According to the compliance rate, the retention rate, and the recruitment rate, the study showed the feasibility of launching the MYmind program for Chinese adolescents with ASD and their parents. The MYmind program in the study achieved an attendance rate of 80%, which was consistent with the findings of 70–100% attendance rate in other MBI studies for ASD individuals and their caregivers (de Bruin et al. 2015; Hartley et al. 2019; Ridderinkhof et al. 2017; Salem-Guirgis et al. 2019). For retention rate, there was no dropout in the MYmind group and the response rate in the post-assessment was 89%. The results were aligned with other MBI studies, which showed a range of 0–40% dropout rates and a range of 60–100% response rates (de Bruin et al. 2015; Hartley et al. 2019; Ridderinkhof et al. 2017; Salem-Guirgis et al. 2019). Apart from the compliance rate and the retention rate, the recruitment rate was adequate and about one-third of the families who registered for the study met our selection criteria. Among the eligible families, nearly 90% of them participated in the study. The majority of the applicants were excluded from the study because they did not meet the inclusion criteria (eg. Age, ASD diagnosis). To conduct a larger RCT in the future, it was suggested to recruit participants from clinics and NGO organizations, which provide service for adolescents with ASD and have a long-term good relationship with the families. Overall, the MYmind program was regarded as comprehensible and acceptable to Chinese adolescents with ASD and their parents, in terms of the easiness of program contents, the helpfulness to parents, and the satisfaction of the program. However, parents perceived the program was helpful to their children to a small extent. To examine the helpfulness of the program in detail, it was suggested to ask both adolescents and parents to rate the usefulness of each training session theme and mindfulness exercises in future studies.

The Holm–Bonferroni sequential correction was used for the analysis of the secondary outcomes. No statistically significant difference was found in within-group or between-group comparison. In the within-group comparison of adolescent’s outcomes, both the MYmind group and the waitlist control group showed improvement in social responsiveness and internalizing problems. The possible explanation for the unexpected improvement in the waitlist control group could be due to the time arrangement of the intervention. As the intervention was arranged during summer vacation, adolescents with ASD did not need to go to school (stress-inducing environment) and therefore might have a lower level of stress, which was associated with improved social functioning and reduced internalizing problems (Bishop-Fitzpatrick et al. 2015; Sheidow et al. 2014). As adolescents with ASD were not under stress, it could be possible that mindfulness demonstrated little effect on the outcomes in the MYmind group. An additional follow-up assessment might be able to see the beneficial effect of mindfulness on adolescents with ASD when school resumed. Furthermore, in the measure of executive function, it was noted that adolescents in the MYmind group had little improvement. It seemed mindfulness had no beneficial effects on executive function of adolescents with ASD. However, as the results were based on parents’ reports and the sample size was small, further investigation of the effectiveness of mindfulness on executive function was suggested. In future studies, self-reported questionnaires and task-based tools are recommended to use as alternative measures of executive function.

In the within-group comparison of parent’s outcomes, the MYmind group had an overall greater improvement than the waitlist control group. However, an increase in laxness parenting was found among parents in the MYmind group, which meant parents tended to act permissively and had fewer demands on their children’s behaviours. This result was in contrast to the previous finding that mindful parenting was negatively associated with laxness parenting (de Bruin et al. 2015; Gouveia et al. 2016). The possible explanation could be due to the cultural difference. As Chinese culture emphasizes order and discipline in parenting, parents in Hong Kong are more demanding and stricter to their children (Shek 1997). They often set high standards and expect their children to follow. As a result, children often suffer from stress and have a lower level of psychosocial well-being (Yip et al. 2019). In the MYmind program, parents were taught to put themselves in their children’s shoes. They learned to accept the limitations of their children, and hence adjusted their expectations and made fewer demands on their children’s behaviours. In future studies, interviews can be included to further understand the effect of mindfulness on parenting style.

In the between-group comparison of adolescent’s and parent’s outcomes, no statistically significant difference was found between the MYmind group and the waitlist control group. Nevertheless, a big between-group effect size was found in the measure of rumination. This suggested that mindfulness might have beneficial effects on reducing the rumination of parents of adolescents with ASD. In regard to prior studies (Deyo et al. 2009; Jury and Jose 2018; Svendsen et al. 2016), positive results were also found that mindfulness was associated with a lower level of rumination. The mechanism could be mindfulness helped individuals acknowledge their thoughts with a non-judgemental attitude, which allowed them to better cope with emotional distress and let go of obsessive thoughts. In addition to rumination, medium between-group effect sizes were found in the measure of mindful parenting (IM-P’s total scale and emotional awareness subscale), parenting stress (parent–child dysfunctional interaction subscale), and parenting style (over-reactivity subscale). Compared to previous studies (Chaplin et al. 2018; Coatsworth et al. 2010; Lo et al. 2017a, b; Potharst et al. 2019), the current results were in alignment; previous results showed there was strong statistical evidence indicating mindfulness might benefit parents of adolescents with ASD by improving these aspects. The mechanism could be explained by the mindful parenting model (Duncan et al. 2009), which proposed that mindfulness helped parents bring emotional awareness to parenting interaction. Parents were therefore able to avoid over-reactive responses to their children and improve the parent–child relationship.

## Limitations

The study had a short follow-up duration; the long-term effects of mindfulness training on adolescents with ASD and their parents were unknown. Also, the study involved a small sample size, which might not be powered enough to detect differences between the MYmind group and the waitlist control group. A bigger sample size was required to accurately measure the effectiveness of mindfulness training on adolescents with ASD and their parents. Furthermore, the study had the following weaknesses: (1) Reliance on parent’s observation to report adolescent’s outcomes. In future studies, additional measures, such as clinical interview, naïve clinician report**,** child self-report, neuropsychological test were suggested to strengthen the results. (2) Only parents filled in the program evaluation. The inclusion of adolescents to fill in the program evaluation could obtain a comprehensive evaluation of the program. (3) The study did not collect any data about whether the adolescents and parents completed weekly homework practice. The lack of practice could be a reason contributing to insignificant results. (4) The reporting timelines of measurements (SRS, CBCL, BRIEF) in the pre-assessment and the post-assessment were overlapped, which might have led to inaccurate results. A longer-term follow-up was suggested to better examine the effects of mindfulness training on adolescents with ASD and their parents. (5) Due to the study design, blinding of the participants and teachers was not possible, but the outcome assessors were blinded to the randomization.

## Conclusion

Due to the increase in ASD prevalence, innovative interventions are needed to support families of children with ASD. This study is the first RCT to investigate the effectiveness of a mindfulness-based intervention (MYmind) on Chinese adolescents with ASD and their parents in Hong Kong, China. Results showed the MYmind program was feasible in the Chinese context. Although the secondary outcomes were not statistically significant, there were some notable findings in parent’s rumination, mindful parenting, parenting style, and parenting stress. Future study with larger sample size and longer follow-up period is suggested to better examine the effectiveness of mindfulness training on adolescents with ASD and their parents.
